# The Interplay between Dynamics and Structure on the Dielectric Tensor of Nanoconfined Water: Surface Charge and Salinity Effect

**DOI:** 10.1021/acs.jpcb.4c05803

**Published:** 2024-11-16

**Authors:** Felipe Mourão Coelho, Luís Fernando Mercier Franco

**Affiliations:** Faculdade de Engenharia Química, Universidade Estadual de Campinas (UNICAMP), Campinas, SP 13083-852, Brazil

## Abstract

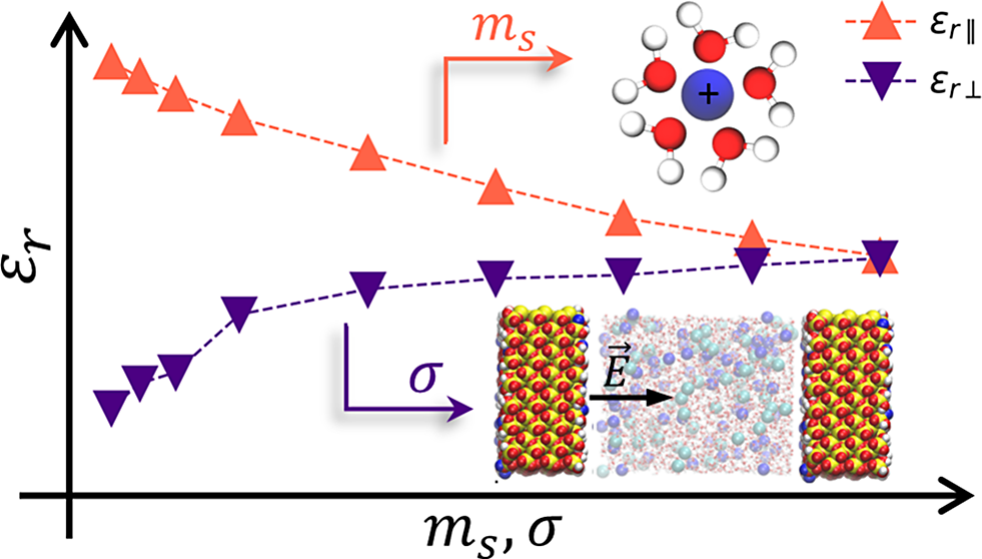

Under confinement, the water dielectric constant is a second-order tensor with an abnormally low out-of-plane element. In our work, we investigate the dielectric tensor of an aqueous NaCl solution confined by a quartz slit-pore. The static dielectric constant is determined from local polarization density fluctuations via molecular dynamics simulations. In a pioneering investigation, we evaluate not only the effect of salinity but also surface charge. The parallel dielectric constant decreases with salinity due to dielectric saturation. From a dynamic perspective, the relaxation of water dipoles is slower within the hydration shells of ions. An anisotropic arrangement on the quartz surface results in preferred orientations of interfacial water molecules. By embedding charge, the surface structure changes, and extra dipole fluctuations in one direction may develop anisotropy in the parallel dielectric constant at the interface. Both surface charge and salinity increase the perpendicular dielectric constant. Nevertheless, the surface charge effect is more pronounced and may even recover the bulk dielectric constant value. The electric field established by the charged surface may disturb the planar hydrogen bond network at the interface, increasing out-of-plane dipolar fluctuations. Our work advances the knowledge of confined dielectric behavior, shedding light on the key role that charged surfaces play.

## Introduction

The solvent’s ability to screen charge and attenuate an external electric field can be quantified by its dielectric constant. Water has strong dipole interactions between molecules, which facilitate collective motion and orientation under an external electric field, resulting in its pronounced relative permittivity (around 80 under ambient conditions^1^). From continuum theory, the permittivity is used to quantify electrostatic interactions and properties of a macroscopic volume of matter in a polarizable solvent.^2^ Certain conditions, such as confinement within micro- and mesopores, locally impact water polarization, and continuum theory may be unsuitable for describing the dielectric constant. Under inhomogeneous media, the dielectric constant is no longer a scalar but a space-dependent second-order tensor.

Close to interfaces, a pronounced solid-like anisotropic arrangement of water molecules is observed. The density is inhomogeneous, and its profile depends on the nature of the surface.^3^ The water hydrogen bond network is altered due to water–substrate interactions, and structural, thermodynamic, and dynamic properties deviate from the bulk phase.^4,5^ A decrease in the melting point temperature, density, and surface tension of water within silica pores is expected.^4^ Even when confined by a highly hydrophobic substrate, the vibrational and infrared spectra may differ substantially.^6^ Under confinement, the phase diagram is dramatically changed, usually with a reduction in the critical temperature, which may occur due to the reduced coordination number of confined molecules.^7^ Transport properties, such as diffusivity, viscosity, and thermal conductivity, are also modified under confinement.^8−12^ All these confinement effects on fluid thermodynamics can be useful for the design of new processes and techniques, for instance, desalination of seawater with graphene and carbon nanotubes^13^

The change in water polarization due to confinement affects the electrostatic interactions intermediated by water molecules, thus modifying hydration and solvation structures.^14^ The dielectric properties of nanoconfined water rule various natural and technological applications. Nanofiltration, reverse osmosis, protein folding, electrophoretic mobility of solutes, seawater desalination in nanochannels, macromolecule–surface interactions, solvation free energy, ion transport in cell membranes, and energy storage in supercapacitors are some examples where the dielectric behavior plays a key role.^15−18^ The development of new nanofluidic devices depends on a comprehensive understanding of the effect of confinement on the water dielectric constant.

Recently, Fumagalli et al.^19^ performed atomic-force scanning dielectric microscopy experiments to investigate water confined by flat crystals of graphite and hexagonal boron nitride. The relative permittivity decreases significantly compared with that of bulk water. For a separation as small as 1.4 nm, an out-of-plane dielectric constant of 2.3 is reported.^19^ In such a scenario, the induced alignment of water dipoles by the surface creates a “dead-layer” of small polarizability molecules. Experimentally, dielectric properties are usually obtained by spectroscopy techniques, which enable the interpretation of spatially averaged properties at the interface.^20^ From the spectral density, the distinction between the dynamics of water in the vicinity of ions and an interface from that in the bulk is not straightforward.^21^

To evaluate the dielectric response of confined systems locally, molecular dynamics (MD) is a powerful tool. In the past, MD simulations have been used to investigate the dielectric constant of water confined by graphite-derived sheets,^2,14−16,22−27^ silica nanopores,^28−32^ diamond,^18^ smectites,^33^ carbon nanotubes,^20^ rutile,^2^ membranes,^3,34^ Lennard–Jones walls,^35^ and soft-matter (decanol).^36^ From atomistic simulations, information at the nanoscale (such as the local relative permittivity and ion-specific potential mean force) can be used to make continuum models (for instance, the extended Poisson–Boltzmann) predict more realistic properties, including double-layer capacitance, electro-osmotic mobility and ionic distribution within the nanopore).^3,37^ Both equilibrium (EMD) and nonequilibrium (NEMD) simulations are suitable to investigate the dielectric response. From EMD simulations, the dielectric tensor may be obtained by linear response theory using principles from the fluctuation–dissipation theorem.^18,38^ In the NEMD framework, an external electric field is applied, and from the change in the system polarization density, the out-of-plane permittivity is determined.^2,26^ Both methods result in the same dielectric response if the linear regime assumption is met.^18,22^ The polarization density may be obtained either by the charge density or by explicitly accounting for the individual multipole density. In the last case, higher-order multiples, such as quadrupoles and octopoles, should also be considered in the determination of the out-of-plane dielectric constant.^18^

The decrease in the perpendicular dielectric constant of water under confinement has also been observed in MD simulations.^2,14−16,22−32,35^ This reduction is usually attributed to the strong alignment of water dipoles close to the surface, which reduces dipole–dipole correlations.^24,25^ In the interfacial region, there may be a higher density of planar hydrogen bonds that constrain the out-of-plane rotation of these molecules.^26^ In a different direction, Olivieri et al.^16^ argued that the lower perpendicular permittivity is unrelated to structural features, but it is actually connected to the low-dielectric confining medium surrounding the fluid and long-ranged anisotropic dipole correlations. Both experiments^19^ and MD simulations^14,22,33^ have shown that the water perpendicular dielectric constant recovers its bulk value only for channel widths in the order of hundreds of nanometers. Similar conclusions are also valid for other protic (methanol and acetonitrile) and aprotic (dichloromethane) solvents.^22^

In the presence of electrolytes, the dielectric constant of water decreases due to dielectric saturation.^21,39^ Water molecules screen the free charge of ions in such a way that their dipoles get partially immobilized in the first and second hydration layers, which decreases their diffusivity^40^ and dipole–dipole correlations.^30^ Under confinement, an anomalous increase in the dielectric constant may occur with electrolytes,^2,15,24,28−30,32^ which has been confirmed recently by experiments.^17^ The presence of salts may disturb the interfacial hydrogen bonds and, through the reorientation of water molecules around the ions, increase the rotational degree of freedom of interfacial molecules and dipole moment fluctuations.^17^ Above a certain critical concentration, the dielectric saturation effect prevails, and the dielectric constant further decreases with the ionic concentration.^28^

In this work, we aim at investigating the dielectric behavior of an aqueous NaCl solution confined by minerals of quartz using equilibrium molecular dynamics simulations. We determine the local tensor of the dielectric constant for a wide range of salinities and surface charges. We show that there may be an interfacial anisotropy not only in the perpendicular direction but also between the parallel directions. We investigate the solution dielectric behavior dynamically from dipole relaxation and how the dielectric tensor is affected by the quartz structure. Finally, we isolate the surface charge effect from salinity to investigate the roles played by each one in the dielectric response.

## Methods

### Dielectric Constant

The dielectric tensor is obtained from equilibrium molecular dynamics simulations assuming that the linear response between a change in the displacement field and a change in the electric field is valid.^38^ From the fluctuation–dissipation theorem, we can express the excess polarization at a vanishing applied electric field.^18^ In a planar system, like a slit-pore with *z* as the confining direction, the dielectric constant (**ε**_**r**_) is a diagonal tensor expressed by their parallel (∥) and perpendicular (⊥) elements:
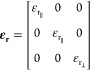
1In the *x* and *y* directions, from Maxwell’s equation (∇ × **E** = 0), the parallel electric field (*E*_*x*_ and *E*_*y*_) is constant.^18^ Therefore, the local parallel dielectric constant may be expressed by
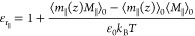
2where *m*_α_ and *M*_α_ are the polarization density and total polarization in the α direction, respectively; ε_0_, *k*_B_, and *T* are the vacuum permittivity, the Boltzmann constant, and the absolute temperature, respectively; and ⟨···⟩_0_ denotes the ensemble average without an external electric field. In an isotropic (ISO) medium, the polarization density is the same in the entire system volume *V* (*m*_α_ = *M*_α_/*V* = **M**/(3*V*)), and the classic Kirkwood fluctuation equation is recovered:
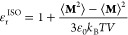
3In the perpendicular direction, the local inverse perpendicular dielectric constant is given by
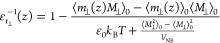
4Our simulations are periodic in all three directions; thus, we also apply Ewald summation in all directions to account for the long-range electrostatic interactions.^41^ The extra term in eq 4—(⟨ *M*_⊥_^2^⟩_0_ – ⟨ *M*_⊥_⟩_0_^2^/)*V*_NB_—is related to the interactions between primary and periodic images in the confining direction. The displacement field has an additional contribution from the periodic images that must be accounted for.^34,36^ Thus, *V*_NB_ stands for the volume of the neighboring periodic image (volume of the fluid plus half of the solid slab). Alternatively, this extra term may be neglected if a large enough vacuum gap is placed between the system in the confining direction, and a pseudo-2D Ewald summation is applied (Ewald3dc^42^). Both approaches should converge to the same result if the long-range electrostatics are appropriately handled.^22^

The polarization density is obtained from the charge distribution in a specific direction. In the perpendicular direction, the polarization density is given by
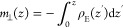
5where ρ_E_ is the total electric charge accounting for both water atoms’ partial charge (bound) and ions (free) charge, and *z* = 0 stands for the solid surface. The parallel polarization density may be obtained by introducing multiple virtual cuts in the perpendicular direction to the respective α-parallel direction (α = *x* or *y*), which split some water molecules, establishing local virtual monopoles with charge density *P*_0_. The polarization density projection in the normal direction of the cut is equal to the surface charge generated by the virtual cut^3,18^ and is given by the average of *N*_C_ cuts in different α positions:
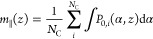
6

Finally, the total polarization in all three directions is obtained by the volume integral of the polarization density:
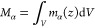
7

For further details on the derivation of the equations and theory, we refer to the work done by Ballenegger and Hansen^38^ and by Bonthuis et al.^18^

We investigate the reorientation dynamics of water molecules by the water dipole moment first-order Legendre polynomial autocorrelation function *C*_μ_(26,43):
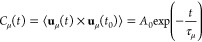
8where **u**_μ_ is a unit vector parallel to the water molecule dipole moment. To improve convergence, multiple time origins *t*_0_ are used, and the autocorrelation function is averaged by each water molecule. The autocorrelation function is fitted to an exponential decay model, and the dipole moment relaxation time τ_μ_ is obtained from the angular coefficient of the plot ln *C*_μ_ (*t*) vs *t*.

### Simulation Details

We investigate the dielectric tensor of an aqueous NaCl solution confined by a slit-pore of quartz (α-SiO) minerals. The quartz structure has 3.4 × 3.4 nm^2^ of surface area, is 1.8 nm thick, has a (100) Miller index, and 9.4 hydroxyls per nm^2^ on the surface.^44^ The confining distance between quartz structures is about 5.1 nm, which is a distance large enough to observe bulk-like features in the parallel directions.^27^ To represent water, the rigid three-point charges SPCE^45^ force field is chosen because it provides an accurate representation of the dielectric constant of liquid water in the bulk phase.^35^ The ions are represented by a classical integer-charge force field developed by Smith and Dang.^46^ CLAYFF^47^ is used to represent quartz. All bonds of the mineral structure are constrained, except for the silanol groups (SiOH), which have a harmonic potential in the angles.

In contact with aqueous solutions, the quartz silanol groups may deprotonate, establishing a negative surface charge. Coelho et al.^11^ fitted a model based on titration experiments^48^ to relate the surface charge with pH and NaCl salinity. We apply this model to determine the surface charge and number of deprotonations necessary to represent each condition and adjust the CLAYFF force field using data from quantum mechanical simulations.^11,49^ Counterions are added in the solution to maintain electroneutrality in the system. The number of molecules and ions placed in the simulation box matches the fluid density in the bulk simulations at the desired salinity. Table S1 of the Supporting Information shows the surface charge and number of molecules for each condition. More details on how the initial system is set up can be found in our previous work.^11^

All simulations are performed in Gromacs.^50^ After energy minimization, the simulations are carried out in the canonical (NVT) ensemble, fixing the temperature at 300 K with a velocity rescaling thermostat.^51^ The convergence of the dielectric function is slow.^16^ By analyzing the evolution of the dielectric response with time, we determine the time from which no meaningful gain in the statistical noise is obtained by increasing the simulation length. Thus, the simulation final time is set at 80 ns with a 2 fs time step. Six independent trajectories are run for each condition to further improve the convergence. The cross-interaction parameters in the Lennard–Jones potential are defined by the Lorentz–Berthelot combining rules, and the cutoff radius for the van der Waals interaction is set to 1.2 nm. The electrostatic interactions in the reciprocal space are handled with the particle-mesh Ewald (PME).^41^ The bonds in the quartz structure and water molecules are kept rigid by the LINear Constraint Solver (LINCS)^52^ and the SETTLE algorithm,^53^ respectively.

## Results and Discussion

**Figure 1:**
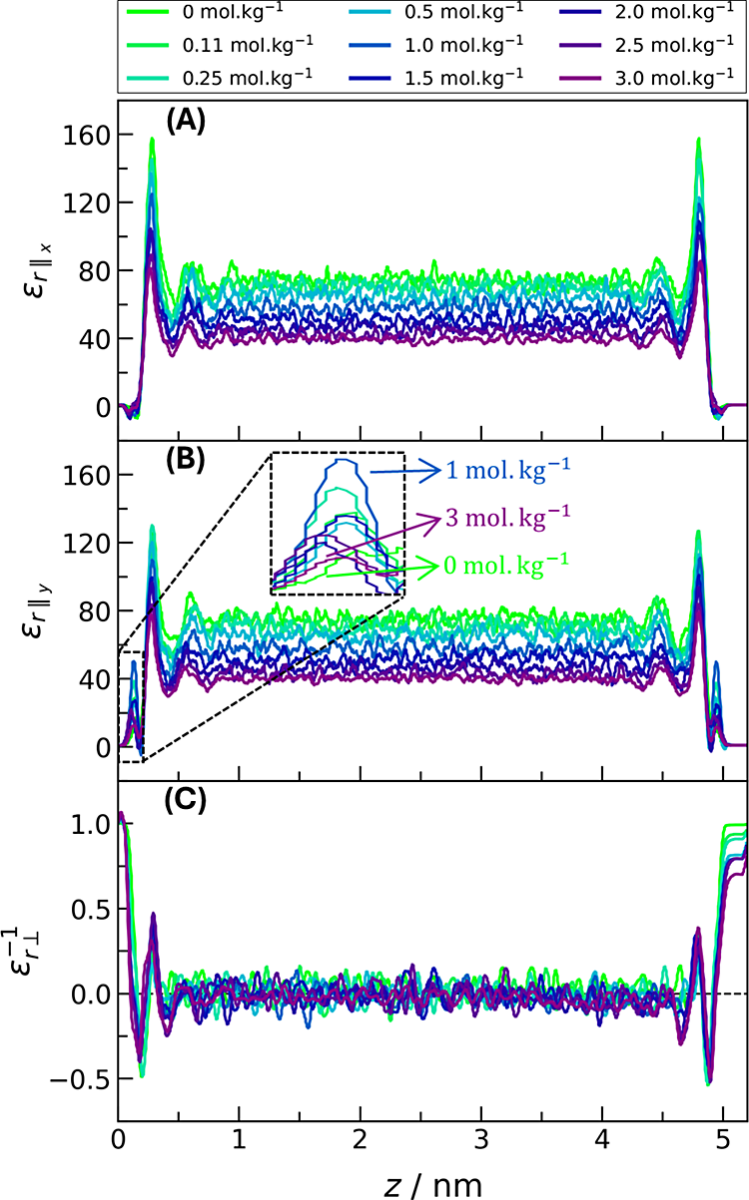
Dielectric tensor within the quartz nanopore in the confined direction at various NaCl molalities. (A) Parallel component in the *x* direction; (B) parallel component in the *y* direction (the inset highlights the first peak); and (C) inverse perpendicular component.

Figure 1 shows the space-dependent dielectric tensors for different salt concentrations. Close to the surface, some oscillations are observed in the dielectric tensor component profile due to the interaction between the surface and interfacial water molecules. Within 1.5 nm < *z* < 3.6 nm, the dielectric tensor achieves bulk-like behavior and is no longer space-dependent. The hydrophilic surface attracts water molecules, increasing the density at the interface and establishing an anisotropic arrangement of molecules. The dielectric constant in an isotropic medium increases with the density (Figure S1a in the Supporting Information). Therefore, due to the higher number of available water molecules, the parallel components of the dielectric tensor (Figure 1A,B) present a peak close to the surface, and the fluctuations follow closely the anisotropic density of water (Figure S1b in the Supporting Information). The proportionality between the confined water density and the dielectric constant (ε_r∥_(*z*) ∝ ρ_w_(*z*)) can be used for a first fast estimation of the relative permittivity in short simulations.^54^ The accuracy of this approach, however, deteriorates in the case of mixtures (local composition may also affect the dielectric tensor) and at the interface of hydrophilic surfaces, which has a pronounced water density but reduced polarization due to the strong interaction between water and the framework.^18^

On the other hand, the perpendicular dielectric constant (Figure 1C) is very low at the interface. In this region, the out-of-plane rotation of water dipoles is constrained, reducing their fluctuations in this direction.^26^ Close to the mineral walls, the negative values of the out-of-plane dielectric constant indicate an overscreening^2,3,26^ caused by the coupling between density and polarization fluctuations.^55^ The overscreening may cause fluctuations locally in the internal electric field, leading to attraction between same-polarity charges and repulsion between opposite-polarity charges at short distances.^55^ A similar situation may occur when the concentration of co-ions locally exceeds the counterions. This phenomenon is called charge inversion, or overcompensation, and is observed in our system at high ionic strength (*m*_s_ > 1 mol kg^–1^).^11^

The dielectric response under confinement depends on the confining media. Bonthuis et al.^18^ investigated the water dielectric tensor confined by two diamond structures: one hydrophobic and one hydrophilic (hydroxyl-terminated). They showed that both parallel and perpendicular dielectric profiles are different after modifying the substrate surface. In this case, the first peak of the parallel dielectric constant is closer to the hydrophilic surface because of its water-wet structure. The peak is higher in the hydrophobic surface because water molecules are weakly bound at this surface.^3^ Thus, the interaction between the confining framework and the fluid has a strong correlation with the local density and polarization of the solvent at the interface, which affects the confined dielectric tensor. The confinement geometry, including the confining shape and distance, also affects the dielectric response. To the best of our knowledge, our work is the first to investigate the dielectric behavior of aqueous solutions confined by charged quartz (full hydroxylated silica). Therefore, a quantitative direct comparison with previous studies is infeasible, and we focus on comparing our results only qualitatively.

**Figure 2:**
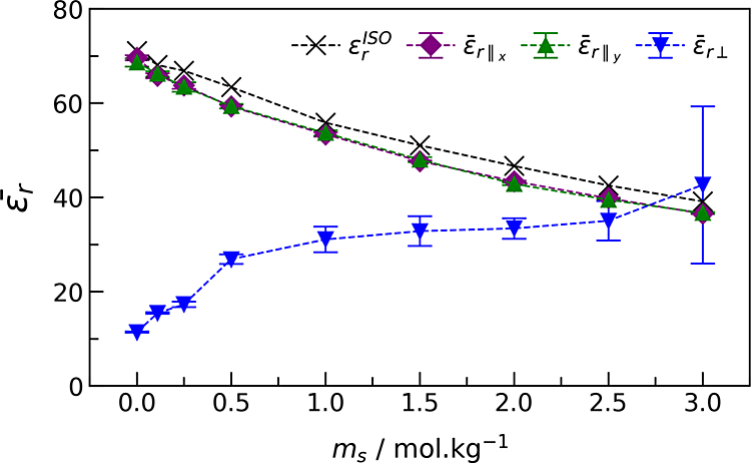
Mean value of each component of the solution dielectric tensor under confinement and in an isotropic medium.

The average value within the mesopore of each component of the dielectric tensor is shown in Figure 2. For comparison, the dielectric constant of the force field in an isotropic medium is also shown.^11^ In the vicinity of electrolytes, water dipoles orient themselves around the ionic charge, decreasing the number of dipole–dipole interactions. Thus, the collective fluid response to attenuate the external electric field is affected, and the dielectric constant decreases (dielectric saturation).^39^ On average, both parallel components are the same and very similar to the isotropic dielectric constant. The difference between them may be attributed to counterions in the confined fluid when *m*_s_ > 0, which may further decrease the medium dielectric constant.

The perpendicular dielectric constant, however, exhibits abnormal behavior: it is very low for pure water, and by adding salt, it increases instead of decreasing, as expected due to dielectric saturation. The gain in water dipole fluctuations from the water molecules reorienting themselves around ions prevails over dielectric saturation in the perpendicular direction for the entire range of salinity, and thus there is no decrease in the out-of-plane permittivity for concentrations above a critical one, as pointed out by Zhu et al.^28^ At higher salt concentrations, the perpendicular dielectric saturation may even recover the bulk value, i.e., the perpendicular element and the isotropic dielectric constant are equal at *m*_s_ = 3 mol kg^–1^ within the statistical error. The larger uncertainties at higher ionic strength are caused by the fluctuations of the inverse of the perpendicular dielectric constant closer to zero. At the interface, the out-of-plane polarization is almost zero due to the surface-induced alignment of water dipoles, resulting in low rotational freedom.^19^ By comparing our system to capacitors in series, wherein , the low mean perpendicular dielectric constant may be attributed to the first water molecule “dead-layer”, i.e., a region with molecules with low mobility and low out-of-plane rotation. Because a harmonic average gives the mean value, the low permittivity at the interface makes a large contribution to the effective out-of-plane dielectric constant, and there may be a confinement effect even for larger pores.^22,25,33^

Although the mean value of the in-plane dielectric constants is the same, we observe anisotropy between the parallel dielectric components at the interface (Figure 1A,B). ε_r∥_*y*__ exhibits a first peak, which does not appear in ε_r∥_*x*__. Such a peak is also dependent on salinity. It is pronounced for intermediate salt concentrations and smaller for pure water or higher ionic strength.

A similar anisotropy in the parallel direction has been reported for confined diffusion coefficients.^56−58^ Franco et al.^56^ investigated the diffusion of methane, nitrogen, and carbon dioxide confined by calcite slit-pores. At the interface, the anisotropy in the parallel self-diffusion coefficients arises from the morphology of the calcite surface. In terms of diffusion, one direction is more favorable than the other, depending on the interplay between the free spaces available in the calcite surface and the interactions between diffusing molecules and ions in the minerals.^56^

**Figure 3:**
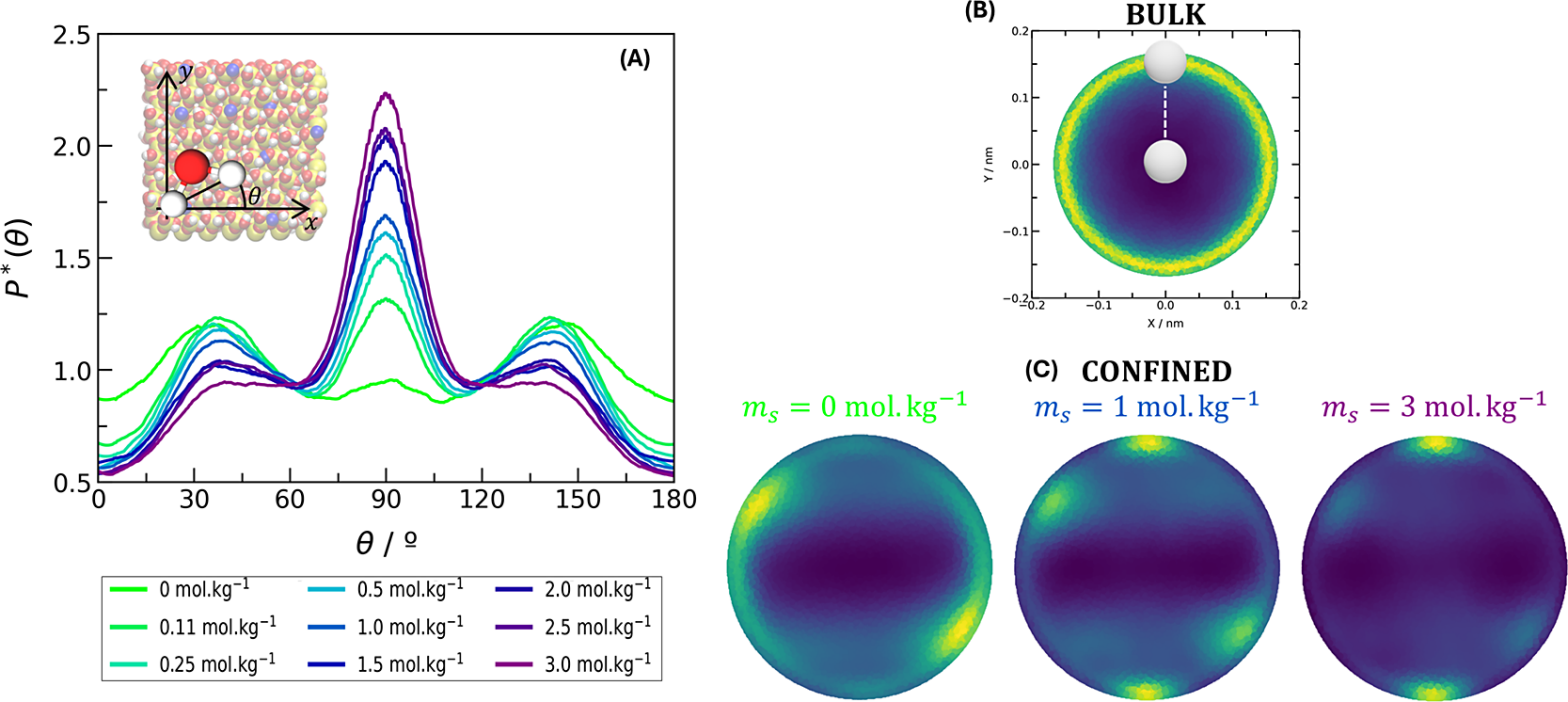
Orientation of water molecules at the interface (*z* < 4 Å). (A) Probability distribution of the angle between the *x*-axis and the projection of the vector  in the *xy* plane normalized by the same probability from water molecules in bulk. Heat map of the  projection in the *xy* plane for (B) bulk and (C) interfacial water molecules. In the heat maps, one hydrogen atom is located in the center, and the color scheme gives the location of the second; yellow points indicate higher probability locations.

To understand the origin of the anisotropy in the parallel dielectric constant, we investigate the structure of interfacial water molecules. In Figure 3, we define the vector , which is the projection in the *xy* plane of the vector connecting the hydrogens atoms from the same water molecule. We, then, analyze the probability distribution of angles between  and the *x*-axis and heat map of the hydrogen atoms in the *xy* plane (Figure 3A). In the bulk, water assumes no specific orientation (Figure 3B). Close to the interface, preferable positions may arise, depending on salinity (Figure 3C). By increasing the salt concentration, a transition in the interfacial water orientation takes place. A diagonal orientation is more common with no salt, whereas at high ionic strength, water hydrogen atoms align vertically along the *y*-axis. At intermediate salt concentrations (for instance, at *m*_s_ = 1 mol kg^–1^), both orientations may be somewhat favorable, in such a way that the transition between states by a water molecule increases dipole fluctuations around the *y*-axis. As a consequence, the first anisotropic peak in ε_r∥_*y*__ is related to the structure assumed by water molecules at the interface.

**Figure 4:**
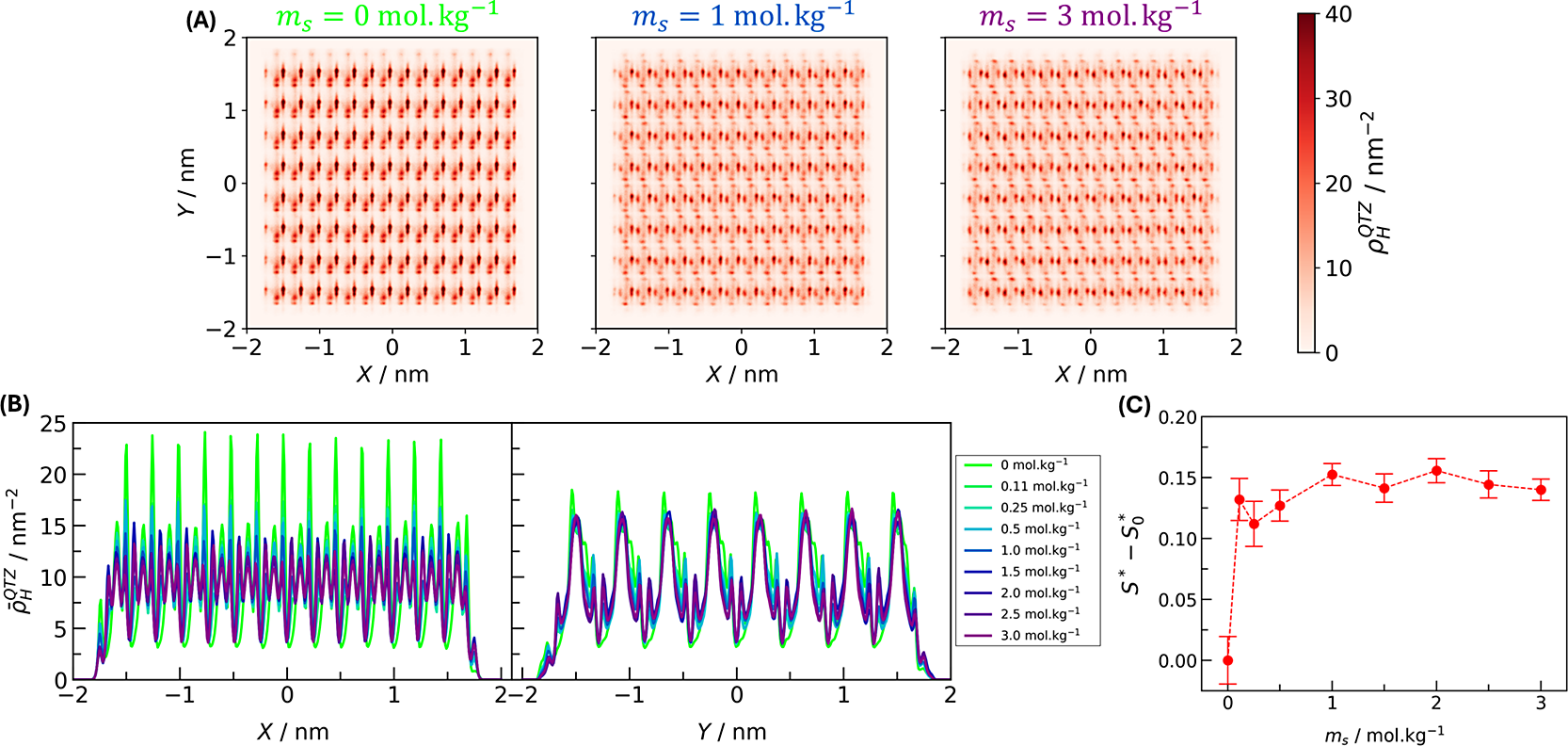
Morphology of the quartz surface for various salinities and surface charges. (A) Surface density heat map of the hydrogen atoms from the silanol groups. (B) Mean surface density of hydrogen atoms at each *x* and *y* direction. (C) Variation of the Shannon entropy of the quartz surface compared to the condition with no salt and no surface charge (*S*_0_^*^).

But why do water molecules change their preferred orientation at the interface depending on the salt concentration? To address this question, we investigate the morphology of the quartz surface. Figure 4A,B shows the surface density of the silanol groups ρ_H_^QTZ^ (here represented by their hydrogen atoms) and its average value ρ̅_H_^QTZ^ in each plane direction, respectively. Figures 3C and 4A show the heat map of the water orientation and the quartz surface, respectively, only for *m*_s_ = 0, 1, and 3 mol kg^–1^. The heat maps for the other conditions are shown in Figures S2 and S3 of the Supporting Information. From the crystallographic plane, the silanol groups are more compact in the *x* direction than in the *y* direction. When there is no salt and no surface charge, the hydrogen atoms of each silanol group are diagonally located from each other. Therefore, the hydrogen atoms from water molecules orient preferably diagonally to the surface, minimizing the interface free energy. At higher ionic strength, the water hydrogen atoms are located closer to the surface, as can be seen in the bound charge density profile in Figure S4 of the Supporting Information. Simultaneously, the hydrogen from the silanol groups becomes more aligned in the *x* direction. Therefore, to once again minimize the repulsion between hydrogen atoms from water and quartz, water molecules orient vertically in the *y* direction, wherein there is more free-space on the quartz surface. In particular, these results and conclusions on the structure of the framework surface may be very dependent on the force field and the surface density of hydroxyl groups. In our investigation, Si–O–H is free to rotate, and we use a fully hydroxylated quartz surface. Ho et al.^59^ showed, for instance, that the interfacial water position and orientation changes with the force field, and that depending on the density of hydroxyl on silica surfaces, the force field also has a great impact on the water contact angle.

The organization of the quartz structure can be quantified by the Shannon’s entropy S*:
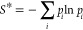
9where *p*_*i*_ is the probability of finding a hydrogen atom in the location *i*, and the sum runs over all possible locations on the quartz surface. The Shannon entropy is a statistical entropy widely used in information theory to measure missing information in a random source.^60^ In our work, we apply the Shannon entropy to gather information about how structured the quartz surface is (Figure 4C). By increasing the salt concentration and surface charge (removing hydrogen atoms), the quartz entropy increases, reaching a plateau around *m*_s_ = 1 mol kg^–1^. Therefore, the quartz surface is more structured with no salt. Otherwise, it has a more sparse distribution of hydrogen atoms. This transition between structures of the quartz surface may cause the reorientation of interfacial water molecules, establishing the anisotropy in the parallel dielectric constant.

The relative permittivity is a static property. Dynamically, the dielectric response may be featured by the dipole moment autocorrelation function. We compute *C*_μ_(*t*) for water molecules within the interfacial region and in the bulk (Figure S5 of the Supporting Information). A characteristic time can be obtained from the autocorrelation function either by integrating it (dipole moment correlation time)^28,31,32^ or by fitting it to an exponential decay (dipole moment relaxation time).^26,27,31^ We show in Figure S6 of the Supporting Information that both approaches result in similar characteristic times for water molecules in the bulk, which indicates that the water molecules behave closely to a Debye dielectric constant in this situation.^31^ In contrast to the bulk behavior, the dipole autocorrelation function does not converge to zero at the interface, resulting in higher discrepancies between the correlation and relaxation times. In this region, a relaxation mode different from that of a Debye relaxation occurs.^31^

**Figure 5:**
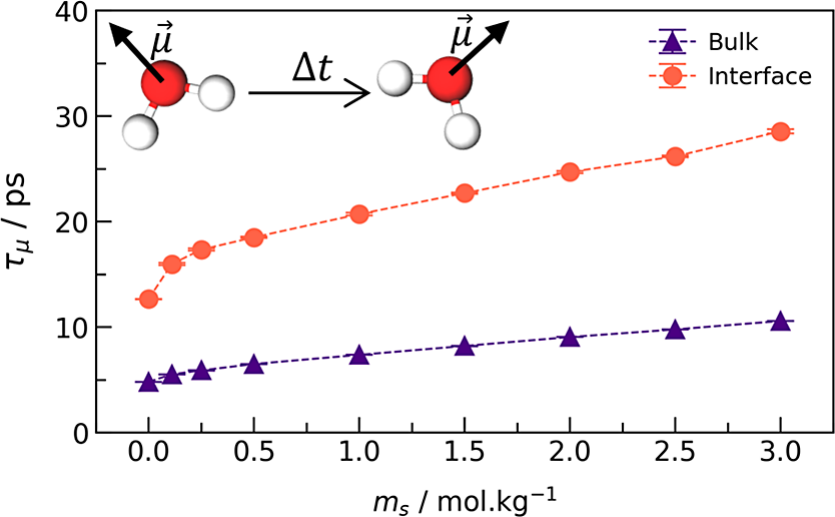
Dipole moment relaxation time of water molecules located at the interface (*z* < 4 Å) and in the bulk (15 Å < *z* < 36 Å) at various NaCl salinities.

Figure 5 shows the dipole relaxation time obtained from fitting the autocorrelation function to an exponential decay model. With ions, the dipole moment takes longer to lose correlation because the water molecules within the hydration shell of ions are trapped and rotationally bonded, screening the ionic charge. From terahertz spectroscopy experiments, Tielrooij et al.^61^ showed that water confined by lipid bilayers present noteworthy lower reorientation dynamics (almost irrotational) at the interface when compared to bulk. Our simulation translates this experimental observation in the dipole relaxation time at the interface, which is significantly higher than that in the bulk. As a consequence, the interface has low polarizability and a low perpendicular dielectric constant.

**Figure 6:**
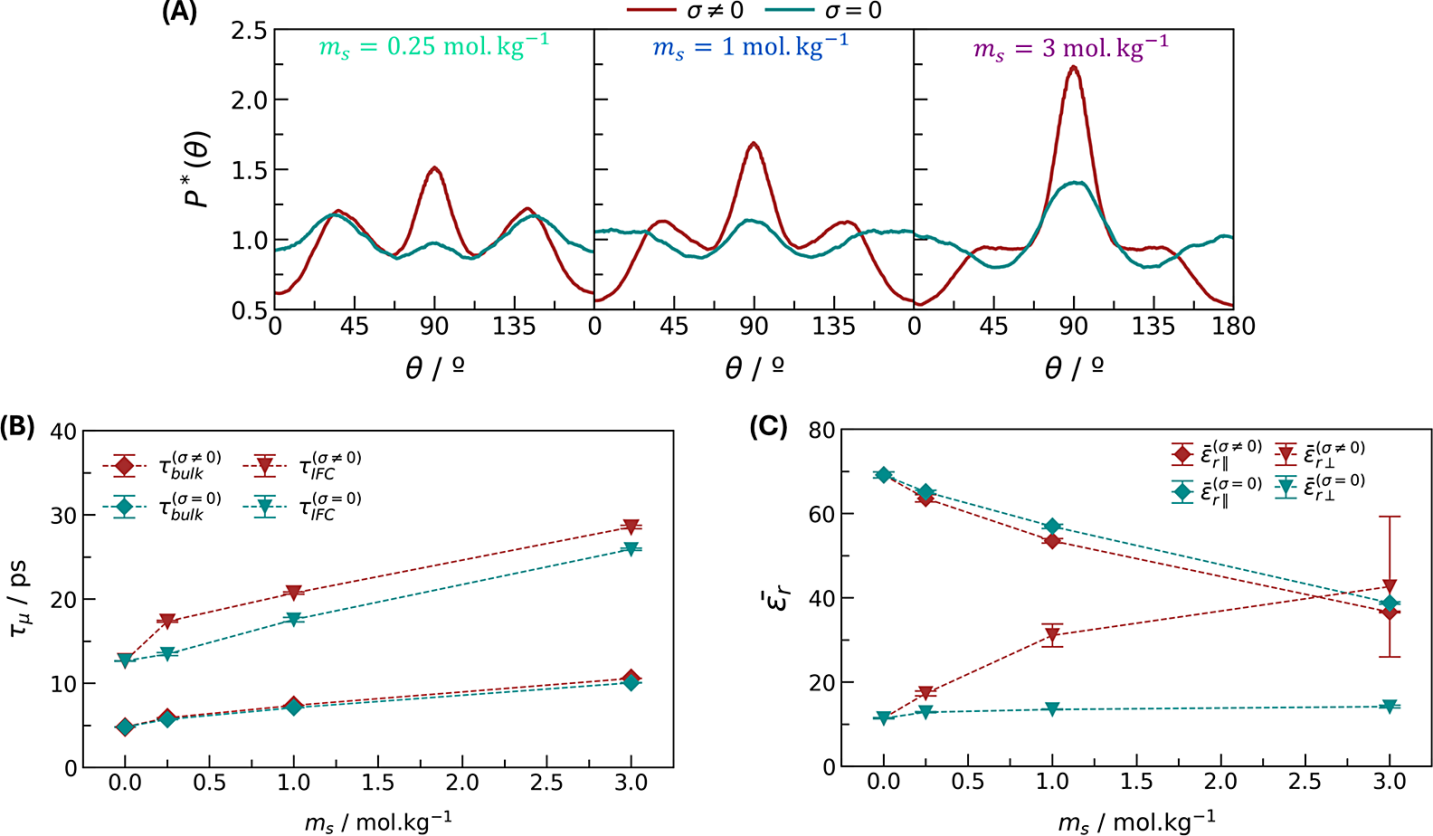
Salinity vs surface charge effect on (A) probability distribution of the interfacial water molecules  orientation, (B) dipole moment relaxation time, and (C) mean parallel and perpendicular dielectric constants. σ = 0 stands for no surface charge, whereas σ ≠ 0 means a salinity-dependent surface charge.

To represent our system realistically, we investigate the effect of salinity coupled with the surface charge (the higher the salt concentration, the higher the quartz surface charge for a certain pH). Nevertheless, molecular dynamics simulations allow us to decouple features to investigate their individual effects. Figure 6 shows some of the properties related to the dielectric response with and without surface charge at the quartz surface. At the interface, water alignment in the *y* direction is more pronounced on charged surfaces (Figure 6A). Otherwise, if the quartz is neutral, there is still some ordering of  vertically due to salt ions, but it is not that different from the random bulk distribution. As a consequence, the anisotropy in the parallel dielectric constant of saline solutions confined by neutral quartz is not that evident, as shown in Figure S7 of the Supporting Information. There is a clear first peak followed by the main one in ε_r∥_*y*__ when charge is embedded on the surface (Figure 1B), whereas both peaks are almost merged if there is no surface charge. From a dynamic perspective, the dipole moment relaxation in the bulk of the pore is the same regardless of whether the surface is charged (Figure 6B). At the interface, the dipole relaxation is slightly faster when the surface is neutral. In this case, the ionic adsorption to the surface is weaker (there are no counterions), and the ionic strength is significantly lower at the interface (Figure S8 of the Supporting Information). Therefore, the water dipole dynamics in each case are mostly affected by the ions in the solution and do not depend directly on the surface structure but rather on the local ionic concentration.

Finally, we computed the mean dielectric tensor at no surface charge (Figure 6C). The parallel dielectric constant (represented by the average between the *x* and *y* directions) shows the same behavior, regardless of the charge on the surface. The dielectric saturation effect, which relates to the relaxation of dipole moments, only depends on the ionic concentration. The small difference between ε_r∥_ values is attributed to the presence of counterions from the charged surface, which further decreases the permittivity. A different scenario occurs in the perpendicular direction. At no surface charge, the out-of-plane dielectric constant only slightly increases with salinity and does not recover the bulk-like dielectric constant. The slight increase may be due to the reorientation dynamics around the ions, which is insufficient to compensate for the confinement effect. Therefore, the abnormal increase observed before is not attributed to the ionic concentration but rather to the surface charge. By charging the quartz surface, an electric field in the confined direction is established, which compensates for the confinement effect imposed by the quartz mineral. With no external electric field, water molecules have a preferred in-plane hydrogen bond network, which reduces the molecules’ out-of-plane reorientation at the interface.^22,26^ With a perpendicular electric field, this planar hydrogen network may be disturbed, increasing out-of-plane dipole moment fluctuations and the perpendicular dielectric constant.

To explore our hypothesis, we perform simulations of pure water confined by charged quartz with the same surface charge as *m*_s_ = 3 mol kg^–1^ (σ = −0.13 C m^–2^). Figure S9a of the Supporting Information shows that the same vertical alignment of interfacial water molecules observed at high salinity occurs if the same charge is embedded in the quartz surface. In terms of the dielectric tensor (Figure S9b,c of the Supporting Information), the parallel component resembles the one from pure water confined by the uncharged quartz with a mean value of ε̅_r∥_ = 64.7 ± 0.4. The perpendicular dielectric constant, however, is closer to that from the saline solution confined by the same charged quartz with an average of ε̅_r⊥_ = 35.5 ± 4.1. Therefore, each component of the dielectric tensor is affected differently. The parallel dielectric constant is most affected by the influence of ions on the dynamics of dipole relaxation, whereas the perpendicular dielectric constant is most affected by the quartz structure and the electric field established by its charged surface.

## Conclusions

We investigate the dielectric response of an aqueous NaCl solution confined by quartz minerals using equilibrium molecular dynamics simulations. From polarization density fluctuations, we obtain the dielectric tensor. The main findings are:

- In the perpendicular direction, confined pure water has an abnormally low dielectric constant, which is attributed to the suppressed dipolar fluctuation in this direction at the interface, caused by the surface-induced alignment of water dipoles. Nevertheless, the out-of-plane dielectric constant increases by both embedding charge in the surface and adding salt to the solution. The increase from the former is dominant. The electric field established by the charged surface may disturb the in-plane hydrogen bond network and increase dipolar fluctuations at the interface. If the surface has enough charge, the mean perpendicular dielectric constant may reach the bulk value.
- The parallel dielectric constant behaves similarly to the bulk dielectric constant: by increasing salinity, the dipolar correlation relaxation becomes slower, and the permittivity decreases due to dielectric saturation. We indicate that there may be anisotropy in the parallel dielectric constant at the interface, related to preferred orientations induced by the structure of the quartz. By embedding charge in the surface, there is a transition in water’s hydrogen atoms from diagonal to vertical. At intermediate salt concentrations and surface charge, both orientations may occur, increasing dipolar fluctuation in the vertical direction and resulting in an anisotropic parallel dielectric constant at the interface.

From our work, we have improved the knowledge on the dielectric behavior of confined salt solutions. Specifically, we explore how structural factors and dynamics affect the dielectric tensor differently, shedding light for the first time on the main role played by the surface charge in the out-of-plane dielectric constant. We have assumed that the linear dielectric response regime is valid. Because our system contains electrolytes and surface charge, this hypothesis may be explored in the future to understand the extension of nonlinear effects on the dielectric response.

## Supporting Information Available

Simulation setup for each condition investigated; density effect on the dielectric constant; heat maps of the orientation of interfacial water and the quartz surface density; bound charge density profile; dipole moment correlation function analysis; and comparison between the effect of surface charge vs salinity (PDF)
