# Supplementary material for: The Interplay between Dynamics and Structure on the Dielectric Tensor of Nanoconfined Water: Surface Charge and Salinity Effect
Source: J Phys Chem B. 2024 Nov 16;128(47):11759–67. doi: 10.1021/acs.jpcb.4c05803 (PMC11613631; doi:10.1021/acs.jpcb.4c05803)
Supplement: Supplementary file 1 — jp4c05803_si_001.pdf [file jp4c05803_si_001.pdf]

SUPPORTING INFORMATION

The Interplay between Dynamics and Structure on the Dielectric Tensor of Nanoconfined Water: Surface Charge and Salinity Effect

Felipe Mourão Coelho and Luís Fernando Mercier Franco*

Universidade Estadual de Campinas (UNICAMP), Faculdade de Engenharia Química, Campinas-SP, 13083-852, Brazil

E-mail: lmfranco@unicamp.br

Simulation Details

Table S1: Simulation setup for each investigated salinity m_s under confinement for pH = 7.2. The number of removed protons ($N_{\mathrm{H_{out}}}$) are based on the model-assigned surface charge (σ) and the number of water molecules (N_w) and salt ion pairs (N_s) is based on the solution bulk density (ρ) at 300 K and 1 bar.

m_s / mol·kg^{-1}	σ / C·m^{-2}	$N_{\mathrm{H_{out}}}$	ρ / kg·m^{-3}	N_w	N_s
0	0	0	998.2	1991	0
0.11	-0.050	4	1002.9	1988	4
0.25	-0.064	5	1008.5	1983	9
0.50	-0.079	6	1018.4	1974	18
1.0	-0.096	7	1037.2	1955	35
1.5	-0.109	8	1055.0	1935	52
2.0	-0.118	9	1071.7	1914	69
2.5	-0.126	9	1087.5	1893	85
3.0	-0.133	10	1102.6	1871	101

Density Effect

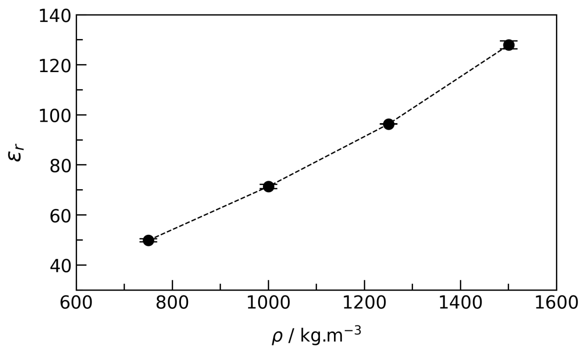

(a)

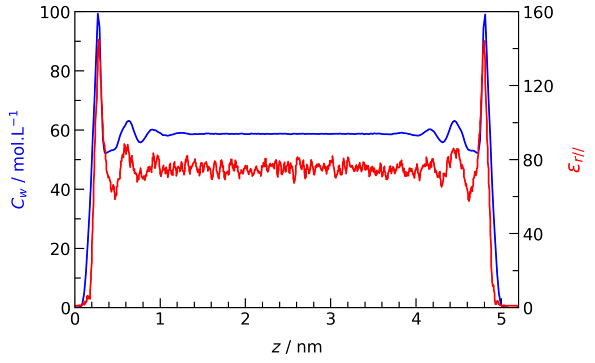

(b)

Figure S1: Density effect on the dielectric constant: (a) dielectric constant of water in an isotropic medium at 300 K; (b) density (blue left axis) *vs.* parallel dielectric tensor (red right axis) profiles of water confined by quartz at 300 K.

Heat Maps

Interfacial Water Orientation

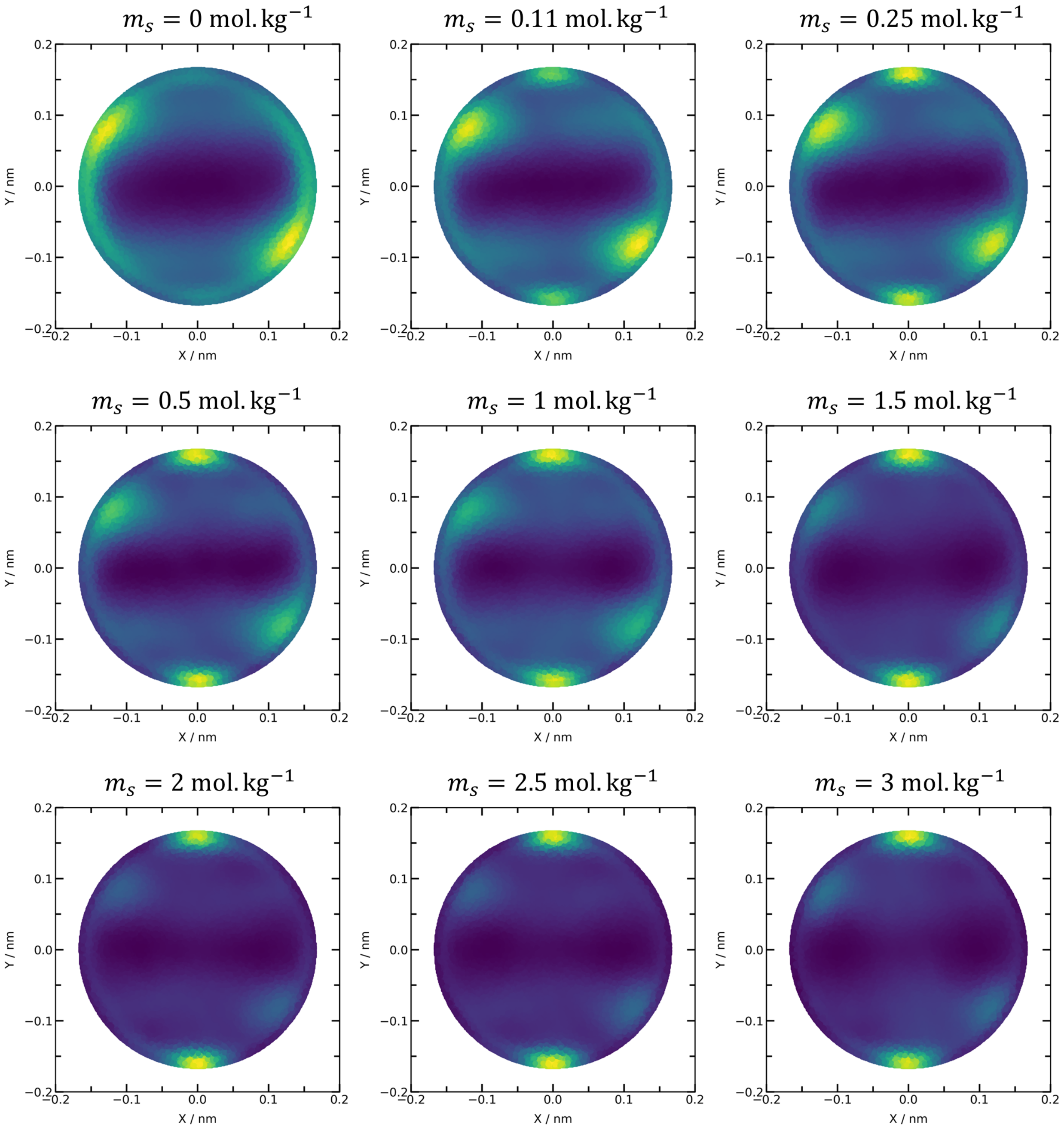

Figure S2: Heat map of the $\overrightarrow{\mathrm{HH}}$ projection in the xy plane for water molecules located close to the quartz interface. In the heat maps, one hydrogen is located in the center, and the color scheme gives the location of the second: yellow points indicate higher probability locations.

Quartz Surface Density

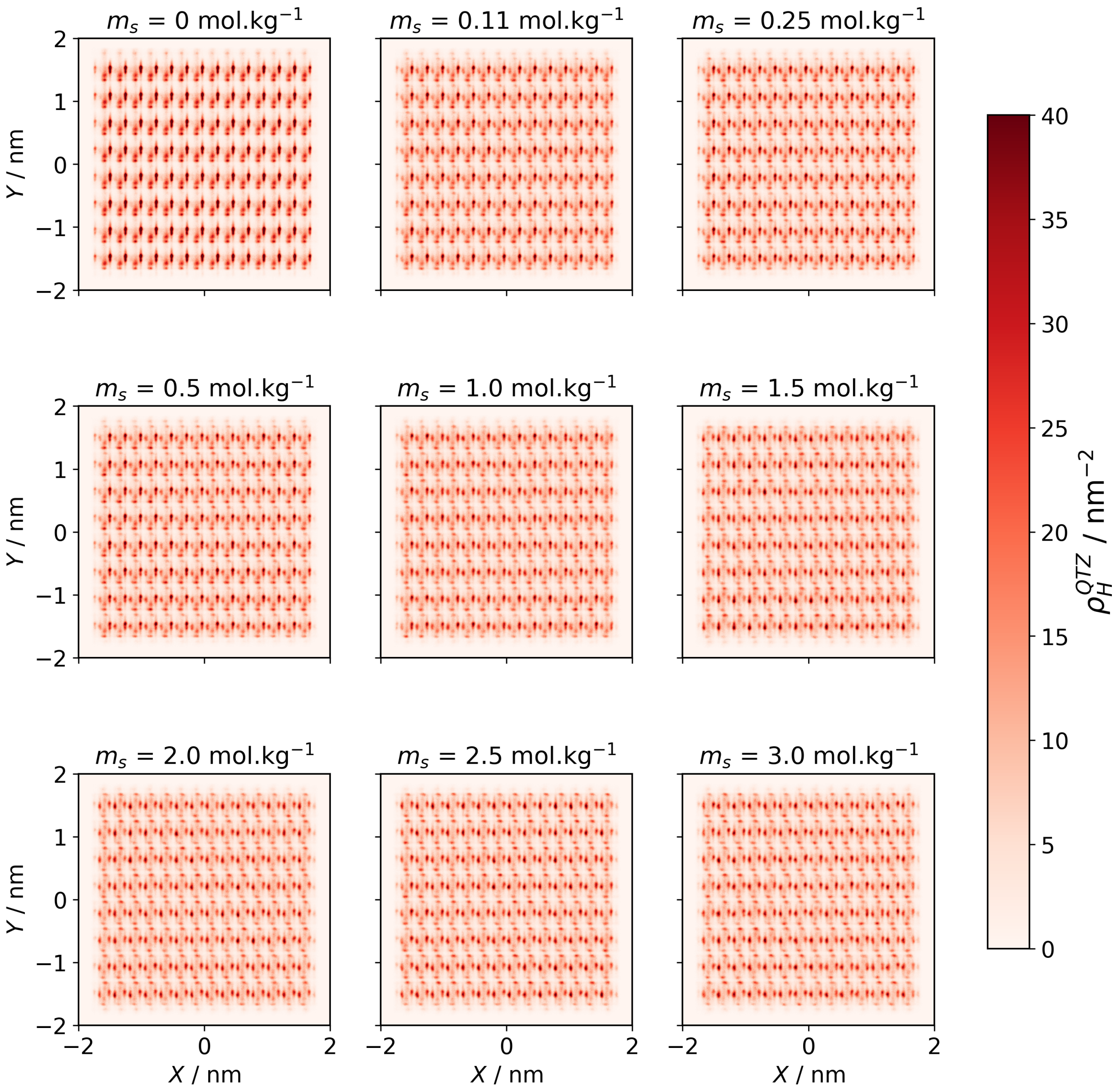

Figure S3: Surface density heat map of the hydrogens from the silanol groups.

Charge Density

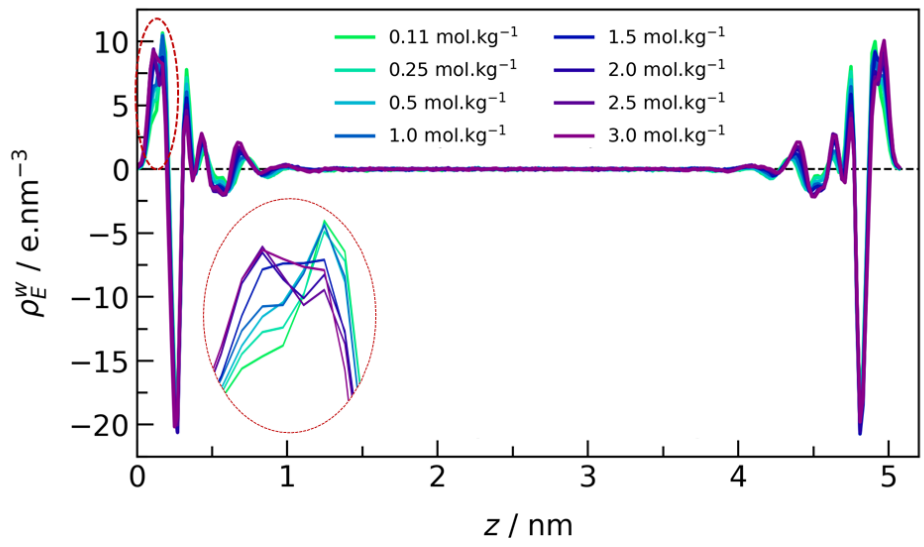

Figure S4: Bound charge density profile from the partial charge of water atoms. The inset highlights the first peak of each salinity.

Dipole Correlation Function

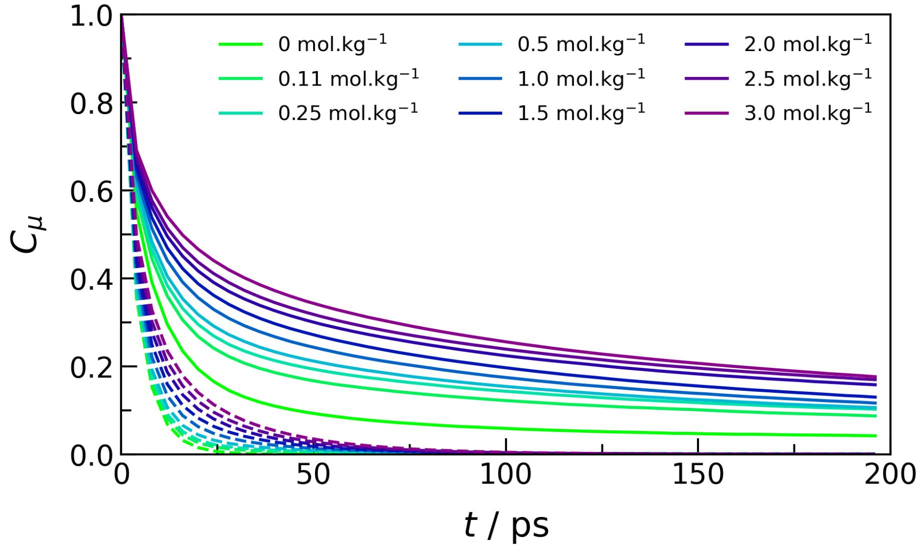

Figure S5: Dipole moment autocorrelation function for various confined NaCl solutions. Dashed lines represent water in the bulk of the pore (15 Å< z < 36 Å), whereas full lines stand for interfacial water molecules (z <4 Å).

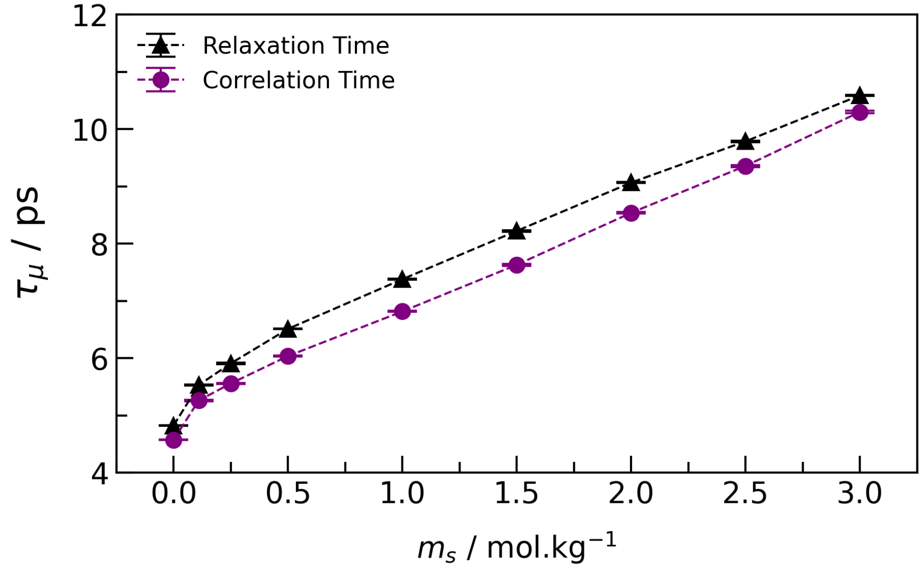

Figure S6: Comparison between the dipole moment correlation time (obtained by integrating the autocorrelation function) and the dipole moment relaxation time (obtained by fitting the autocorrelation function to an exponential decay model) for water molecules in the bulk of the pore (15 Å< z < 36 Å).

Surface Charge Effect

Dielectric Tensor

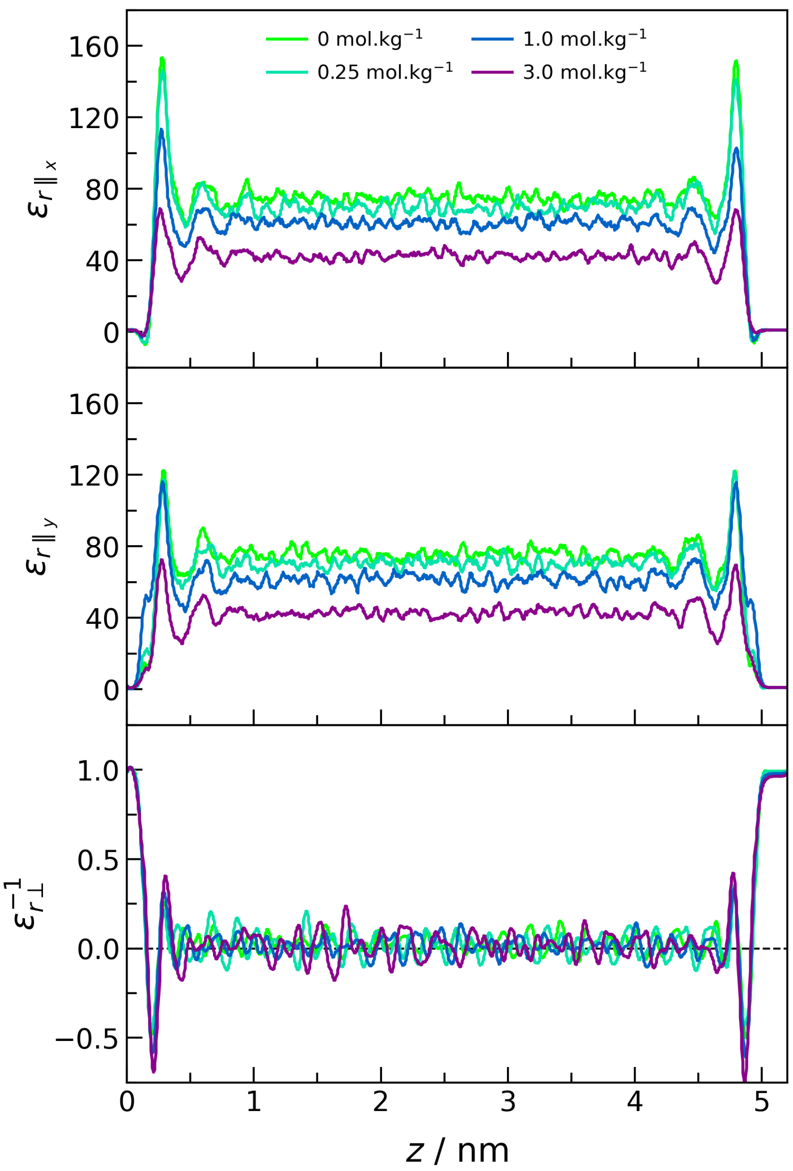

Figure S7: Dielectric tensor within the uncharged quartz nanopore ($\sigma = 0$) in the confined direction at various NaCl salinity. The upper, middle, and lower plots represent, respectively, the parallel component in the x direction, the parallel component in the y direction, and the inverse perpendicular component. The salt concentration legend in the upper plot applies to the others.

Ionic Distribution

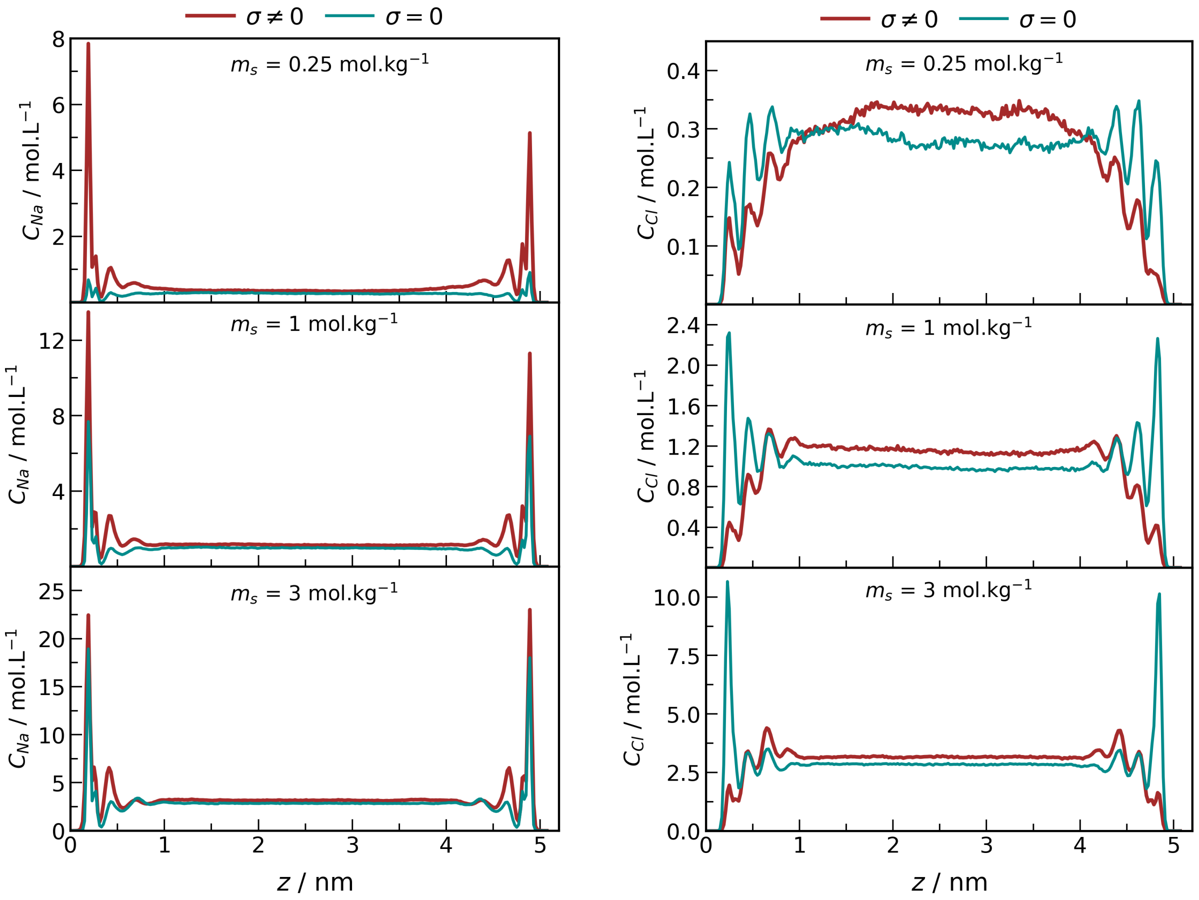

(a) (b)

Figure S8: Ionic distribution within the charged ($\sigma \neq 0$) and uncharged ($\sigma = 0$) quartz nanopore for various salt concentrations: (a) Na^+ cations and (b) Cl^- anions density profiles.

Salt Concentration *vs.* Surface Charge

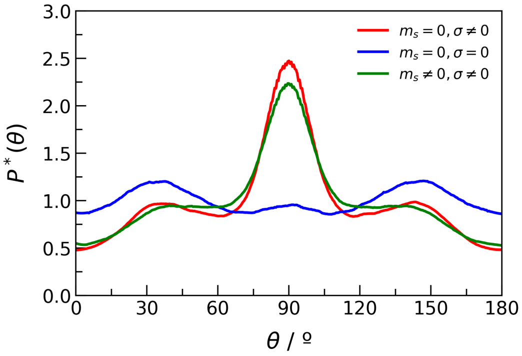

(a)

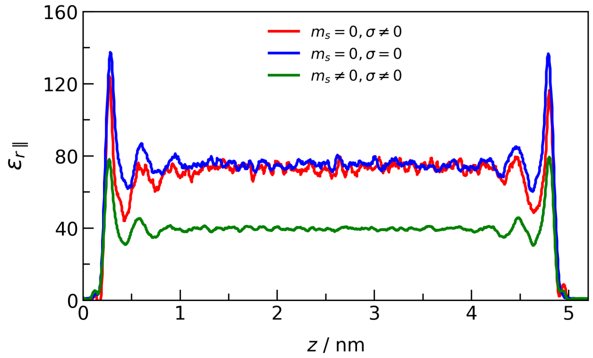

(b)

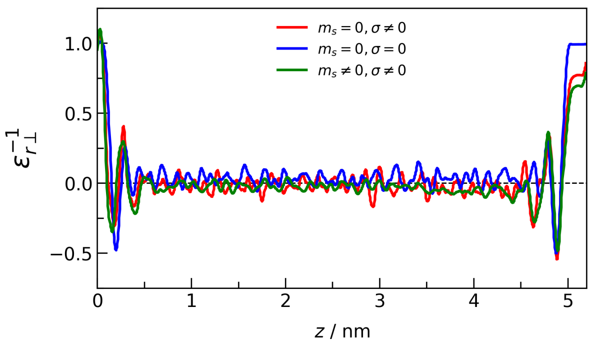

(c)

Figure S9: Effect of salt concentration and surface charge on (a) the probability distribution of the interfacial water molecules ($\overrightarrow{\mathrm{HH}}$) orientation, (b) parallel dielectric constant, and (c) perpendicular dielectric constant. The surface charge ($\sigma \neq 0$) and salt concentration ($m_s \neq 0$) are -0.133 C.m^{-2} and 3 mol.kg^{-1}, respectively.
